# Families, Friends, and the Neighborhood of Older Adults: Evidence from Public Housing in Singapore

**DOI:** 10.1155/2012/659806

**Published:** 2011-11-22

**Authors:** Treena Wu, Angelique Chan

**Affiliations:** Health Services & Systems Research, Duke-NUS Graduate Medical School, Level 4, 8 College Road, Singapore 169857

## Abstract

*Introduction*. This empirical paper examines how the Housing Development Board (HDB) public housing neighborhood influences older urban Singaporeans' social interactions and ameliorates social isolation. *Methods*. Using 4,542 observations of noninstitutionalized urban adults aged 60 and above, ordered logistic regressions are run to determine the predictors of isolation while controlling for physical health and demographics. *Results*. 87% of older Singaporeans reside in public housing apartments while 13% reside in private market housing. The main predictor of social isolation is living alone and the second main predictor is coresidence with adult children. The relationship between coresidence with adult children and isolation is mediated when controlling for older adult functional limitations. The public apartment neighborhood and daily participation in public neighborhood events have substantial effects on reducing the risk of isolation. Older adult contact with friends alleviates isolation more than contact with non-coresiding relatives. *Conclusion*. Findings suggest that the public neighborhood-built environment in Singapore plays a positive role in the social interactions of the elderly. Knowledge of the factors that decrease the risk of social isolation will have implications for studying morbidity and mortality among the elderly.

## 1. Introduction

In newly industrialized economies (NIE) in Asia, economic growth and demographic changes are leading to longer life and smaller family size. In Asia, given the Confucian beliefs of filial piety, it has been traditionally expected that the younger members of the family provide time, money, goods, and instrumental and emotional support for older adults. However, with later marriage and lower fertility, it is now more likely that older adults in Asian NIE have smaller families and a higher likelihood of living alone. As a consequence elders in urban settings may have fewer social interactions as they age. However it is not necessarily the case that late life is characterized by social isolation.

Individuals can adapt to the aging process with changes in their behavior and the environment. Older adults may compensate for a loss of social interaction when their families become smaller by interacting more with friends and neighbors who are in close physical proximity. This may especially be the case for the oldest old with functional limitations who may not only be neighborhood-based but neighborhood-bound. We hypothesize that in the Singapore densely populated city state the built neighborhood environment contributes to older adult social interactions and ameliorates social isolation. Using state Housing Development Board (HDB) policy, we examine social interactions in the neighborhoods of older urban Singaporeans aged 60 and above.

Scholars studying the aging process have wrestled with the assumption that, in later life, an individual gradually disengages from society and inevitably becomes isolated [1]. However, it is posited that an older adult can choose to adapt the size of the social network and the quality of each contact. The composition and the extent of the social network of the spouse, children, friends, and neighbors can possibly change and be refined; this can vary for the young old and oldest old. To test our hypothesis, we are guided by the psychology theory of socioemotional selectivity by Carstensen et al. [2].

Most studies of social relationships in later life focus on the amount (e.g., number of individuals, frequency of contact) or content (practical help, advice) of social contact, not on individuals' perceived social isolation [3]. Isolation is often linked to a higher risk of worse health such as the risk of all-cause mortality, increased morbidity, depression, and cognitive decline [4]. Subjective interpretations of social relationships are likely to be a key to understanding the impact of actual social connections on older adult health and well-being.

Based on the social-psychology disengagement theory of aging by Cumming and Henry [1], there is decreased interaction between the aging individual and others in the social system; but when disengagement is complete, the equilibrium which existed in middle age between the individual and his society will give way to a new equilibrium characterized by a greater distance and an altered type of relationship. This altered state is arguably isolation where the aged individual may be without any form of support. In contrast, socioemotional selectivity theory [2] states that as resources and energy decline in late life, older adults shed less intimate or rewarding relationships and increase their emotional investments in relationships that are more intimate or rewarding. In applying this theory to social networks, we argue that older Singaporeans can be motivated to selectively and actively engage with others in the social system. Disengagement and withdrawal from society in late life may not necessarily occur.

There are several aspects of social network connectedness that contribute to the ease of late life transitions and a lessening of isolation. One of these aspects is the number of direct ties to people and where some types of social ties may be more beneficial than others. There is the value of ties with kin members, who are likely to provide unconditional instrumental and emotional support [5, 6]. Another aspect is the value of close-knit social contacts in which the older person's contacts in a network know each other. This makes for a social network that enables the older adult's contacts to provide instrumental and emotional support, share care-giving duties, and pool resources.

For older adults especially the oldest old, due to debilitating health problems, neighbor interaction and neighborhood attachment may play a large role in their social networks. Older adults may be more vulnerable to the influence of their residential environment as they tend to travel outside their own neighborhoods less often than do younger adults and children who travel for work and school and tend to have a longer duration of exposure to neighborhood influences than younger individuals [7]. Being neighborhood-bound can then affect the older person's perceptions of the neighborhood. In a British cross-sectional population survey of people aged 65 plus living at home, Bowling and Stafford [8] find that individual perceptions of the area as neighborly and having good facilities are independently associated with lower likelihood of low social activities. Within the context of the city in the US, Subramanian et al. [9] find that a neighborhood with residential stability and a concentration of elders is positively associated with older person self-rated health.

## 2. Methods

We carried out an ordered logit estimation using the Singapore Social Isolation, Health, and Lifestyles Survey (SIHLS) 2009 cross-sectional data of 5,000 noninstitutionalized urban Singaporeans aged 60 and above. The SIHLS provides information on the older adult's extent of social isolation; health status and well-being; income, social engagement, housing, network support, and loneliness. The nationally representative survey data was collected using face-to-face interviews with older adults. Almost 90% of Singaporeans reside in HDB public apartment housing. The remaining 10% with higher household incomes reside in private housing.

The state agency HDB was established in Singapore to provide guarantees of housing for its citizens. The unchallengeable right to housing in the densely populated city state was achieved through the construction of affordable urban public housing which began in 1960. Public housing works started in 1960 when Singapore was still a British colony. In 1965, Singapore achieved Independence. From the 1980s, the HDB shifted its focus to building communities within self-contained towns. In spatial terms, because of close proximity, each HDB apartment building has become a neighborhood block; a cluster of neighborhood blocks has become an urban community equipped with social support services for the elderly and children and public spaces such as playgrounds, markets, and cafés, all with the aim of building a sense of place and community [10]. As life expectancy now for men is approximately 79 years and for women 84 years [11], there are increased interventions that promote aging-in-place such as day care and home care support services at the ground level of a HDB apartment block; apartments for the elderly with activities of daily living (ADL) limitations retrofitted with alarm buttons for emergency assistance; communal living for the oldest old without spouses or children; state subsidized senior activity centers that provide organized group activities. Based on HDB population level household survey interviews [12, 13] with residents, it is found that the longer the residence in the same neighborhood block and community, the greater the sense of belonging. This is especially for residents aged 55 and above with a length of residence that is 10 years or more. Thus, the older adult is likely able to maintain an intimate social network or build a stronger social network of family, friends, and neighbors.

HDB manages the public housing stock. This consists of approximately 90% of the total housing stock in the market. The monthly income of the household head that is below SGD$8,000 (Singapore Dollar $1 = US$0.81) and the family size form the criteria of housing assignment to a given apartment. But this rule does not apply to the secondary or resale market. On the basis of this income threshold, each family is then allocated to a HDB apartment building block which consists of different built-up area sizes. Within each HDB neighborhood block, there is variation in household income from the lowest monthly income group of <SGD$500 to the SGD$8,000 threshold. The lowest income group resides in one-room HDB apartments, and the highest income group resides in four-room or five-room HDB apartments. Adult children starting their own homes have preferential access to an apartment that is in close physical proximity to their parents' apartment, which can then enable frequent contact.

Beyond this SGD$8,000 monthly income threshold, individuals then purchase housing from the private housing stock which makes up the remaining 10% of the total stock. While higher socioeconomic status individuals aspire to switch from public housing to private housing, the vast majority of individuals particularly the younger age groups upgrade from smaller size HDB apartments to five-room HDB apartments [10]. The private housing stock consists of condominiums (gated communities with security and key card access), private apartments, bungalow houses, semidetached houses, terrace houses, and townhouses. Geographically, public housing and private housing are mixed because of land shortage in the island state. Public apartment buildings can be located next to private apartment buildings. However, public housing and state subsidized social activity centers are geographically concentrated. The care support services and social activity centers are within close walking distance for the elderly in public housing.

### 2.1. Data

Table 1 shows a description of the key variables that we used from SIHLS such as the outcome variable social isolation; residential type, composition, and size of the social network; age and covariates including physical health and demographics.

For the outcome variable of perceived isolation, respondents were asked “How often do you feel isolated from others?” This subjective measure is on a scale of 1–5, 1 = lowest level, and 5 = highest level. In our analysis, we study the social network as an interaction between the social network and feelings of isolation change. As physical health is an age-related factor, we wanted to control for physical health and assess whether the association between the social network and isolation holds. The physical health measures that we used were difficulties with activities of daily living (ADLs) which refer to self-care tasks and instrumental activities of daily living (IADLs) which refer to the ability to carry out activities associated with maintaining a household. For ADL limitations, respondents were asked the number of difficulties they had with the following activities: (1) take a bath/shower, (2) dress up, (3) eat, (4) transfer stand up from a bed/chair, (5) walk around the house, (6) walk outside of the house, (7) use a squatting toilet, and (8) use a sitting toilet. For IADL limitations, respondents were asked the number of difficulties they had with the following activities: (1) prepare own meals, (2) leave the home to purchase necessary items or medication, (3) take care of financial matters such as paying utilities, (4) use the phone, (5) light housework, (6) take public transport to leave home, and (7) take medication as prescribed.

### 2.2. Empirical Specification

The aim is to understand how social interactions taking place within the HDB neighborhood environment may decrease isolation. The relationship between the HDB-built environment and perceived isolation may be operating through the older adult's perception of the neighborhood. Bowling and Stafford [8] found that individual perceptions of the area as neighborly and having good facilities are independently associated with lower likelihood of low social activities. In our empirical specifications, we included the explanatory variable of daily participation in HDB neighborhood events as a proxy for neighborhood perception.

Using our cross-sectional data, we first start by gaining an understanding of the distribution of social interactions with non-coresiding kin members and nonkin members. We would like to understand how the elderly choose to interact with social contacts outside of the home and how this changes with age. If contacts outside of the home are intimate and rewarding relationships, we hypothesize that the elderly will make more of an effort to stay connected. We then compared this with the distribution of social interactions for older adults in private housing. For these distributions, we used locally weighted bivariate regressions and we did not control for health. From these bivariate regressions, we then explored in depth the composition of the social network consisting of family within the home and non-coresiding relatives, friends, and neighbors outside of the home using multivariate regression.

Multivariate regression techniques were used for generating estimates of perceived isolation. We first regressed the measure of perceived isolation on the variables, HDB residence, participation in HDB neighborhood events, household size, whether the older adult is widowed, whether the older adult lives alone or coresides with a child/children, and age. The focus is on how the older adult's relationships with the family within the home and residence area vary with isolation. The explanatory variable, participation in HDB neighborhood activity is a proxy for the older adult's neighborhood perception. If the older adult perceives the neighborhood favorably, then there is a high likelihood of participation in social activity within the neighborhood. Because of data limitations we were unable to add a variable for length of residence in the same neighborhood.

We then proceeded to factor in the social network of non-coresiding relatives including children, friends, and neighbors who are outside of the home. The social connectedness of non-coresiding relatives and friends was specified as the interaction between the number of individuals connected and the frequency of contact each month. Types of contact include face-to-face visits in the home and outside of the home and phone calls. We did not specify such an interaction for neighbor connectedness because of data limitations. The covariates used were physical health, income, gender, and ethnicity. Physical health is an age-related factor in terms of functional limitations that deteriorate with age. We present a likelihood ratio test to determine if the covariates make a difference to the outcome when not included.

## 3. Results

**Figure 1:**
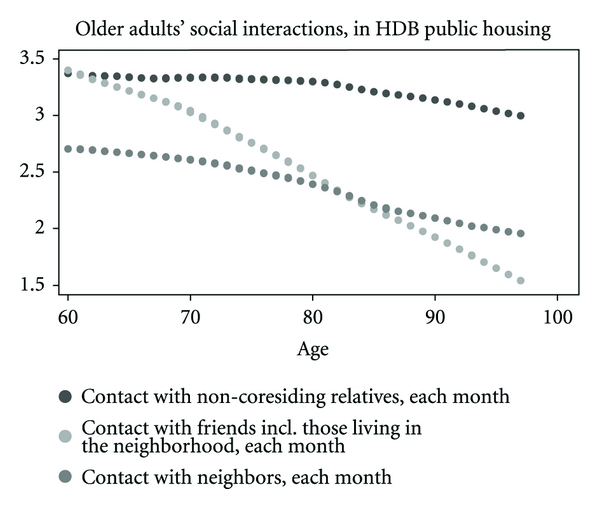
These locally weighted bivariate regressions do not control for health.

**Figure 2:**
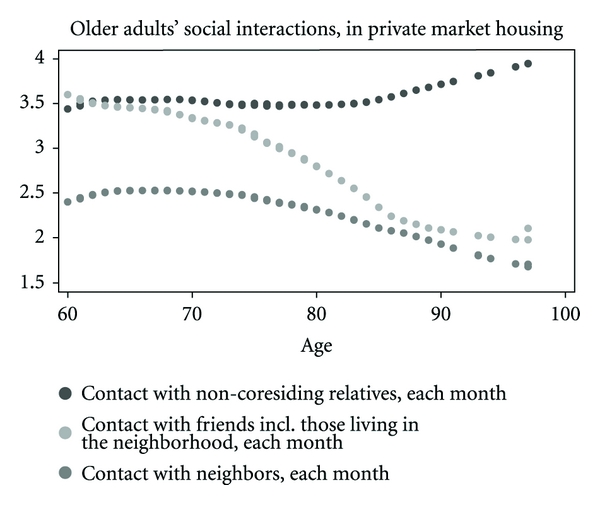
These locally weighted bivariate regressions do not control for health.

Using kernel regressions, Figures 1 and 2 show the age for distributions of social interaction among non-coresiding relatives, friends, and neighbors by HDB housing and private market housing. They provide useful information on the pattern of social interactions outside of the home which is important to consider for the elderly with physical mobility that deteriorates with age. Contact with non-coresiding relatives remains at relatively high levels across age. A decline of contact with friends and neighbors starts within the range of age 70 and 80, and the decline is sharpest for contact with friends, including those who live in the same neighborhood. The decline is sharper for those in a HDB neighborhood than for those in private housing. However, Figure 2 shows that for individuals from the mid 80s to 100, contact with neighbors in HDB housing falls at a slower rate than contact with friends. The oldest old in public housing appears to substitute neighbors for friends.

From Table 1, mean age of respondents is 72.8 years and 54.9% of them are women. 12.7% report suffering from isolation occasionally, fairly often, or always. 35.5% are widowed, and 5.8% live alone. 87.3% reside in HDB public housing, and 78.1% report daily participation in HDB neighborhood events. 76.7% report that they have monthly contact with three or more relatives. 58.2% report that they often, fairly often, or always see or hear from close relatives. 67.5% report that they have monthly contact with three or more friends including friends who live in the same neighborhood. 46.6% report that they often, fairly often, or always see or hear from friends. 56.9% report that they have monthly contact with three or more neighbors who may not necessarily be friends.

Table 2 provides the predictors for perceived social isolation using ordered logistic regressions. The predictors are expressed as coefficient effect sizes, *β*. The first model (1) does not include the social network outside the home or covariates. (2), (3), (4), and (5) include the social network outside the home and covariates. The different contacts that make up the social network are added stepwise across (3), (4), and (5). In the first model, (1) which excludes the social network outside the home, the strongest predictor of isolation is living alone (*β* = 0.683, *P* < 0.01). Similarly when household size is smaller, there is a higher likelihood of perceived isolation (*β* = −0.165, *P* < 0.01). From this model the second strongest predictor of isolation is whether the older adult coresides with adult children (*β* = 0.332, *P* < 0.01). However, when physical health and demographic controls were added, the effect of coresidence with adult children on increasing isolation weakens (*β* = 0.247, *P* < 0.05).

In terms of whether isolation increases with age, we found a relatively small positive effect (*β* = 0.011, *P* < 0.05) in the first model (1). The effect size of age remains very small even when the social network is incorporated and even after controlling for physical health, income, and demographics. See models (2), (3), (4), and (5).

Next, we focused on predictors of lower isolation. From model (1) in Table 2, older adult residence in HDB public housing ameliorates the likelihood of perceived isolation (*β* = −0.169, *P* < 0.05). The HDB coefficient effect size remains strong and statistically significant across models (2), (3), (4), and (5). We attempt to explain the relationship between HDB neighborhood and isolation through older adult daily participation in HDB neighborhood social activities via models (4) and (5) when contact with friends is added. From (4), daily participation in social activities in the HDB neighborhood has a substantial effect on lessening isolation (*β* = −0.164, *P* < 0.05). There is a similar coefficient effect size in (5). From (4) controlling for other variables, contact with friends has a positive effect on lessening isolation (*β* = −0.035, *P* < 0.01). There is a similar coefficient effect size in (5).

## 4. Discussion

The results show that the strongest predictor of isolation in old age is living alone. Unexpectedly, the second main predictor of isolation is coresidence with children. This result is somewhat surprising as it is traditional Singaporean practice for older adults to coreside with children and to some extent grandchildren. The positive association between coresidence with adult children and isolation has some similarity to studies on the determinants of older adult subjective well-being. In a review of sociological and psychological studies on aging and well-being, George [14] finds that interacting with adult children appears to have a weak or nonexistent relationship with subjective well-being. But from our findings, the relationship between living with adult children in old age and isolation is mediated when there is consideration for the older adult's functional limitations. The extent of ADL and IADL limitations may increase their dependency on coresiding children for instrumental support and assuage perceived isolation.

The strongest predictors for decreasing the likelihood of isolation are in order, residence in HDB public housing and daily social participation in HDB neighborhood events. The elderly are very likely to view their HDB neighborhood favorably because of the availability of social care and support services and public spaces for social interaction. The HDB built environment may then be perceived as conducive for social contact for the elderly who are neighborhood-based and neighborhood-bound. In contrast, growing old in private condominiums which are gated communities or bungalow houses that are fenced off may inadvertently create a sense of being cut off from society.

The social network of non-coresiding relatives and the elders' friends also has influence on reducing isolation. In comparing the relationship between the different social contact types, contact with friends has a far more positive effect on alleviating isolation compared to contact with non-coresiding relatives including children. Contact with neighbors does not have any effect on perceived isolation. From the literature on elderly subjective well-being, George [14] summarizes studies that show that friends are generally more important for subjective well-being in later life than are relationships with children or other relatives. Friendships that are sustained in late life may be more intimate as the elderly grow old together and reminisce about the rapid modernization of Singapore over the span of 46 years.

Following the application of socioemotional selectivity theory to social networks, we have provided some understanding about the predictors for reduced social isolation among Singaporean elderly. The HDB neighborhood environment plays a positive role in the social interactions of the elderly who are neighborhood-based and neighborhood-bound. Knowledge of the factors that decrease the risk of social isolation will have implications for studying morbidity and mortality in old age. But the cross-sectional nature of the data limits our analyses in that we are unable to directly assess how individuals transition into late life and how behavioral adaptation and isolation vary as the young old become the oldest old. Also, we are unable to make clear arguments for causal relationships or to fully distinguish between age and cohort effects.

## Figures and Tables

**Table 1:** Description of variables.

Variable	Description	
Social isolation	“How often do you feel isolated from others?”	Never = 56.6%, Rarely = 30.7%, Occasionally = 9.5%, Fairly Often = 2.3%, Always = 1%

Residence in HDB housing	The respondent resides in HDB public housing	Yes = 87.3%, No = 12.7%

Daily participation in a HDB neighborhood event		Yes = 78.1%, No = 21.9%

Network Composition and size	The respondent is widowed	Yes = 35.5%, No = 64.5%
	The respondent lives alone	Yes = 5.8%, No = 94.2%
	The respondent lives with children	Yes = 69.6%, No = 30.4%
	“If you live with your children, what is the household size?”	Mean = 4.2, SD = 1.6
	“Among all your relatives not living with you (including children and grandchildren), how many relatives do you see or hear from at least once a month?”	0 relatives = 14.7%, 1 relative = 4.1%, 2 relatives = 12.5%, 3-4 relatives = 26.9%, 5–8 relatives = 23.9%, ≥9 relatives = 17.9%
	“Among all your friends including those who live in your neighborhood, how many friends do you see or hear from at least once a month?”	0 friends = 14.7%, 1 friend = 3.6%, 2 friends = 14.1%, 3-4 friends = 30.7%, 5–8 friends = 14.9%, ≥9 friends = 22%
	“Among all your neighbors including those you consider your friend, how many neighbors do you see or hear from at least once a month?”	0 neighbors = 11.1%, 1 neighbor = 10.1%, 2 neighbors = 21.8%, 3-4 neighbors = 36%, 5–8 neighbors = 12.4%, ≥9 neighbors = 8.6%

Frequency of Contact within Network	“How often do you see or hear from relatives with whom you have the most contact?”	Never = 5%, Seldom = 11.6%, Sometimes = 25%, Often = 36.9%, Very Often = 12.5%, Always = 9%
	“How often do you see or hear from friends including those who live in your neighborhood with whom you have the most contact?”	Never = 13.2%, Seldom = 12.6%, Sometimes = 27.7%, Often = 32.8%, Very Often = 7.7%, Always = 6%
	There is no question in the survey on the frequency of contact with neighbors including those considered as friends	

Age		Min = 60, Max = 101 Mean = 72.8, SD = 8.1

Income	Household monthly income (Singapore Dollar $1 = US$0.81)	Less than S$500 = 9.5%, S$500–S$999 = 14.6%, S$1,000 to S$1,999 = 24.6%, S$2,000 to S$2,999 = 15.9%, S$3,000 to S$3,999 = 6.2%, S$4,000 to S$4,999 = 2.8%, ≥S$5,000 = 3.2%, refuse to respond = 3.2%, do not know = 20%

Physical health	Self-rated difficulties with the following eight ADLs: (1) take a bath/shower, (2) dress up, (3) eat, (4) transfer stand up from a bed/chair, (5) walk around the house, (6) walk outside of the house, (7) use a squatting toilet, and (8) use a sitting toilet	0 ADL difficulty = 62.7% 1 ADL difficulty = 13.6% 2-3 ADL difficulties = 12.7% ≥4 ADL difficulties = 11%
	Self-rated difficulties with the following seven IADLs: (1) prepare own meals, (2) leave the home to purchase necessary items or medication, (3) take care of financial matters such as paying utilities, (4) use the phone, (5) light housework, (6) take public transport to leave home, and (7) take medication as prescribed	0 IADL difficulty = 82.5% 1-2 IADL difficulties = 8.5% ≥3 IADL difficulties = 9%

Gender	The respondent is female	Female = 54.9%, Male = 45.1%

Ethnicity		Chinese = 71.52%, Malay = 17.08%, Indian = 10.22%, Other ethnicities = 1.18%

**Table 2:** Marginal effect coefficients from ordered logistic regression models predicting older adult perceived social isolation.

	(1)	(2)	(3)	(4)	(5)
Residence in HDB	−.169** (.086)	−.162* (.087)	−.186** (.087)	−.209** (.088)	−.219** (.088)
Daily participation in HDB neighborhood events	.008 (.006)	−.114 (.070)	−.100 (.070)	−.164** (.071)	−.160** (.071)
Living arrangements					
Household size	−.165*** (.022)	−.140*** (.022)	−.138*** (.022)	−.139*** (.022)	−.139*** (.022)
Widowed	.137* (.071)	.210** (.079)	.200** (.079)	.197** (.080)	.199** (.080)
Residing alone	.683*** (.134)	.680*** (.134)	.615*** (.135)	.652*** (.135)	.655*** (.135)
Coresiding with adult children	.332*** (.082)	.247** (.083)	.248** (.083)	.234** (.084)	.230** (.084)
Social network					
Relatives incl. non-coresiding children			−.023*** (.004)	−.010** (.005)	−.012** (.005)
Friends				−.035*** (.005)	−.037*** (.005)
Neighbors					.040 (.024)
Age	.011** (.004)	.003 (.004)	.003 (.004)	−.0007 (.0044)	−.0006 (.004)
Covariates	No	Yes	Yes	Yes	Yes
					
Observations	4,542	4,542	4,542	4,542	4,542

Notes: ***P < 0.01, **P < 0.05 and *P < 0.10. The covariates are physical health limitations—ADL and IADL, income, gender, and ethnicity. The likelihood ratio test for the restricted model without the covariates and the unrestricted model with covariates shows that there is no variation in the main coefficients of interest—HDB residence, daily participation in HDB neighborhood events, family living arrangements, and the social network. LR Chi^2^(4) = 336.02. Prob > Chi^2^ = 0.0000.

## Appendix

See Tables 1 and 2
